# Longitudinal CSF Findings in Autoimmune Encephalitis—A Monocentric Cohort Study

**DOI:** 10.3389/fimmu.2021.646940

**Published:** 2021-03-22

**Authors:** Tobias Zrzavy, Romana Höftberger, Isabella Wimmer, Thomas Berger, Paulus Rommer, Stefan Macher

**Affiliations:** ^1^ Department of Neurology, Medical University of Vienna, Vienna, Austria; ^2^ Division of Neuropathology and Neurochemistry, Department of Neurology, Medical University of Vienna, Vienna, Austria

**Keywords:** autoimmune encephalitis, CSF, oligoclonal bands, pleocytosis, auto-antibodies

## Abstract

Autoimmune encephalitis (AIE) poses a diagnostic challenge due to its heterogeneous clinical presentation, which overlaps with various neurological and psychiatric diseases. During the diagnostic work-up, cerebrospinal fluid (CSF) is routinely obtained, allowing for differential diagnostics as well as for the determination of antibody subclasses and specificities. In this monocentric cohort study, we describe initial and serial CSF findings of 33 patients diagnosed with antibody-associated AIE (LGI1 (n=8), NMDA (n=7), CASPR2 (n=3), IgLON5 (n=3), AMPAR (n=1), GAD65/67 (n=4), Yo (n=3), Ma-1/2 (n=2), CV2 (n=2)). Routine CSF parameters of 12.1% of AIE patients were in normal ranges, while 60.6% showed elevated protein levels and 45.4% had intrathecal oligoclonal bands (OCBs). Repeated CSF analyses showed a trend towards normalization of initial pathological CSF findings, while relapses were more likely to be associated with increased cell counts and total protein levels. OCB status conversion in anti-NMDARE patients coincided with clinical improvement. In summary, we show that in routine CSF analysis at diagnosis, a considerable number of patients with AIE did not exhibit alteration in the CSF and therefore, diagnosis may be delayed if antibody testing is not performed. Moreover, OCB status in anti-NMDAR AIE patients could represent a potential prognostic biomarker, however further studies are necessary to validate these exploratory findings.

## Introduction

Autoimmune encephalitides (AIEs) are an inflammatory disease spectrum affecting the central nervous system (CNS) and can be difficult to diagnose accurately and promptly due to their different clinical presentation (1, 2). Within the last decades, a large number of auto-antibodies with either direct pathogenic effects or implications of yet unknown significance have been discovered and associated with different clinical and neuropathological entities in the spectrum of AIEs (3).

Early immunotherapy is associated with better outcomes, especially when patients mount an antibody response against surface antigens in contrast to antibodies binding to intracellular antigens (4, 5). However, diagnostic assessment for the suspected antibodies can take up to several weeks and is not accessible everywhere, which may delay diagnosis and start of immunotherapy.

For the diagnosis of patients with suspected AIE, the cerebrospinal fluid (CSF) is thoroughly tested, amongst others also for auto-antibodies. However, classical analyses provide more timely information. Parameters such as cell count, intrathecal immunoglobulin synthesis, oligoclonal bands (OCBs) and protein levels indicate inflammatory processes and help to classify disease aetiology as well as to make therapy decisions (6). In this context, pleocytosis and intrathecal immunoglobulins are included in the diagnostic criteria of AIE (1). CSF parameters appear to be variable at the onset of disease and are likely to be antibody subtype-specific; however, certain profiles have not yet been clearly established and CSF alterations might also be influenced by age (7). Besides, only sparse data on longitudinal changes in routine CSF analysis is available; however, it could provide major insights into the dynamics of the disease.

The aim of this monocentric study is to report CSF analyses of AIE patients associated with known auto-antibodies both at onset of disease and during disease course.

## Material and Methods

### Ethics

This retrospective study was approved by the ethics committee of the Medical University of Vienna (EK 1773/2016; 1123/2015).

### Study Population

This is a retrospective analysis of patients recruited at the Department of Neurology, Medical University of Vienna between 2014 and 2020. Patients fulfilling the criteria for AIE (1, 8) were eligible after exclusion of any other potential differential diagnosis, e.g. infectious encephalitis. Only patients who had undergone a lumbar puncture (LP) were included. A total of 42 patients were screened and 33 patients were included in the final analyses. 9 patients had to be excluded due to missing data about the first LP, concomitant viral infection or antibody overlaps. Auto-antibodies (abs) were determined using a well-established in-house routine cell-based-assay as reported earlier (9). If more than 5 LPs were performed, only LPs with relevant changes were reported.

### CSF

CSF analyses were performed at the Division of Neuropathology and Neurochemistry, Department of Neurology, Medical University of Vienna, a laboratory certified by the Austrian national accreditation body. The following CSF parameter were analyzed according to routine procedures and protocols: cell count per µL (upper reference limit: ≤ 4 cells/µL), total protein (upper reference limit: ≤ 40mg/dl) as well as levels of albumin in the CSF (upper reference limit: ≤ 35 mg/dl). Simultaneously, albumin levels were measured in the serum as well and the *Q*_Alb_ (serum/CSF) was calculated. The upper limit of *Q*_Alb_ was calculated as 4 + (*α*/15) with *α* representing the patient’s age (10). Immunoglobulins (IgG, IgM, IgA) and albumin were measured using immunonephelometry. Isoelectric focusing of paired CSF and serum samples was used to identify CSF‐specific oligoclonal IgG bands (OCB).

### Statistical Analysis

Descriptive statistics were used for presentation of clinical data and CSF parameters. Categorical variables were expressed in frequencies and percentages. Parametric continuous variables were expressed as mean and min-max; nonparametric variables as median and range.

### Data Availability

Anonymized data can be made available upon reasonable request from a qualified investigator after approval of the ethics review board of the Medical University of Vienna.

## Results

### Cohort Characteristics

A total of 33 patients were included in this longitudinal study. Detailed demographics are shown in **Table 1**. The mean age was 50.6 years. The male to female ratio was 13 to 20 and the median time from first symptom to LP was 136 days. Psychiatric symptoms were the most common clinical features observed at the very beginning of the disease. The most common cell-surface-antibody-associated AIEs (sAIE) were anti-LGI1 (8/22), followed by anti-NMDAR abs (7/22). Anti-GAD-abs (4/11) were the most frequent group of intracellular-antibody-associated AIEs (iAIE). In 16 of the 33 patients, follow-up LPs (2-5; min-max) were performed between 10 and a maximum of 2360 days after the first LP.

**Table 1:** Demographic and clinical characteristics of the entire cohort at the time of diagnosis.

	sAIE		iAIE	
Age at onset^1^	49.36	(19.74)	53.27	(15.2)
Females^2^	10	(45)	10	(90)
Time from onset to LP^3^	62.50	(0-1778)	379.00	(15-2284)
Antibody^2^				
LGI1	8	(36)		
NMDAR	7	(32)		
CASPR2	3	(14)		
IgLON5	3	(14)		
AMPAR	1	(4)		
GAD65/67			4	(36)
Yo (PCA1)			3	(27)
Ma-1/2			2	(18)
CV2 (CRMP5)			2	(18)
Symptoms at onset^2^				
Psychiatric	10	(45)	2	(18)
Cognitive	10	(45)	1	(9)
Seizure	8	(36)	1	(9)
Movement	5	(23)	9	(82)
MRI abnormalities^2^	17	(77)	4	(37)
Tumor^2^	4	(18)	7	(63)

^1^mean and standard deviation ^2^number (percentage) ^3^median and min-max in days; NMDAR, N-methyl-D-aspartate receptor; LGI1, leucine-rich glioma-inactivated 1; CASPR2, contactin-associated protein-like 2; AMPAR, amino-3-hydroxy-5-hydroxy-5-methyl-4-isoxazolepropionic acid receptor; ANNA, anti-neuronal nuclear antibody; CRMP, collapsin response-mediator protein-5, sAIE cell-surface antibody-associated AIE; iAIE antibodies against intracellular antigen-associated AIE.

### CSF Findings at Onset

The overall median cell count was 4 cells per µL (range 0-115). Pleocytosis was present in 45.4% of all patients. 64% of patients with iAIE showed elevated CSF cell counts, whereas only 36.6% of patients with sAIE displayed pleocytosis, with the highest cell counts in patients in the anti-NMDAR encephalitis (anti-NMDARE) subgroup (**Table 2**).

**Table 2:** CSF findings at first lumbar puncture.

	CC^1^		TP^1^		Alb^1^		$Q_{Alb}^{1}$		IgG^2^		IgA^2^		IgM^2^		OCB^3^	AB C^3^	AB S^3^
NMDAR	45	(40,5)	42,4	(19,3)	23,8	(17,9)	5,96	(3,71)	3	(0;6,5)	1	(0;0,5)	0,0	(0;0)	5/7	7/7	5/7
LGI1	2,83	(2,5)	43,4	(16,5)	29,7	(12,1)	6,84	(3,69)	1	(0;0,1)	0,0	(0;0)	0,0	(0;0)	1/8	7/8	8/8
CASPR2	2	(1,5)	35,1	(29,7)	23,4	(22,1)	5,8	(8,3)	0,0	(0;0)	0,0	(0;0)	0,0	(0;0)	0/3	2/3	3/3
IgLON5	2,3	(1)	45,7	(5,1)	29,6	(2,9)	7,5	(0,5)	0,0	(0;0)	0,0	(0;0)	0,0	(0;0)	0/3	3/3	3/3
AMPAR	2	(0)	40,4	(0)	26,5	(0)	5,3	(0)	0,0	(0;0)	0,0	(0;0)	0,0	(0;0)	0/1	1/1	0/1
GAD65/67	12	(3)	69,1	(7,5)	29	(11,1)	4,2	(2,6)	1	(0;0,7)	0,0	(0;0)	0,0	(0;0)	2/4	4/4	3/4
Yo (PCA1)	9	(4)	34,5	(4,6)	21,7	(5,8)	5,6	(1,4)	2	(0;0,9)	0,0	(0;0)	0,0	(0;0)	3/3	3/3	3/3
CV2 (CRMP5)	35,5	(28,5)	75,5	(18,2)	37,9	(2,2)	10,1	(0,6)	1	(0;22,8)	0,0	(0;0)	0,0	(0;0)	2/2	2/2	2/2
Ma-1/2	7,5	(5,5)	81,3	(35,7)	50,3	(27,6)	13,8	(5)	1	(0;1,8)	0	(0)	0,0	(0)	1/2	2/2	2/2

^1^median and range; ^2^number of patients with intrathecal IG synthesis and range; ^3^number of positive patients; CC, cell count per µL; TP, total protein in mg/dl; Alb, Albumin per mg/dl; Q_Alb_ Quotient Albumin serum/CSF; IgG intrathecal IgG mg/dl; IgA intrathecal IgA mg/dl; IgM intrathecal IgM mg/dl; OCB, oligoclonal bands; AB C: antibody detection CSF; AB S: antibody detection serum.

60.6% patients of our total cohort (63.3% in sAIE and 54.5% in iAIE) showed an elevated total protein content (TP) with a mean concentration of 46.1 mg/dl (range: 18.4 – 116.9). Albumin quotients (*Q*_Alb_) were elevated in 7 out of 22 patients in the sAIE group and in 2 out of 11 iAIE patients.

Positive OCBs (IgG) were identified in 45.4% of our total cohort. The highest frequency of positive OCBs was found in patients with anti-NMDARE (5/7) and anti-Yo AIE (3/3). None of the patients with anti-CASPR2-antibodies and only one of the patients with anti-LGI1-antibodies revealed positive OCBs. Intrathecal Ig synthesis was observed in 5/11 iAIE and in 4/22 sAIE patients. Quantitative measurement of intrathecal immunoglobulins in the CSF showed that the predominant Ig isotype observed in this cohort was IgG with one case of IgA and no evidence of intrathecal production of IgM. 12.1% of patients with AIE exhibited normal cell counts in addition to normal *Q*_Alb_, TP levels and absent OCBs.

### Serial CSF Findings

Serial CSF analyses were available in 16 of 33 patients, mainly within the sAIE group. From these 16 patients, data from a total of 54 LPs were available (mean of 3 LPs per patient). Mean duration between consecutive CSF analyses was 356 days.

**Figure 1:**
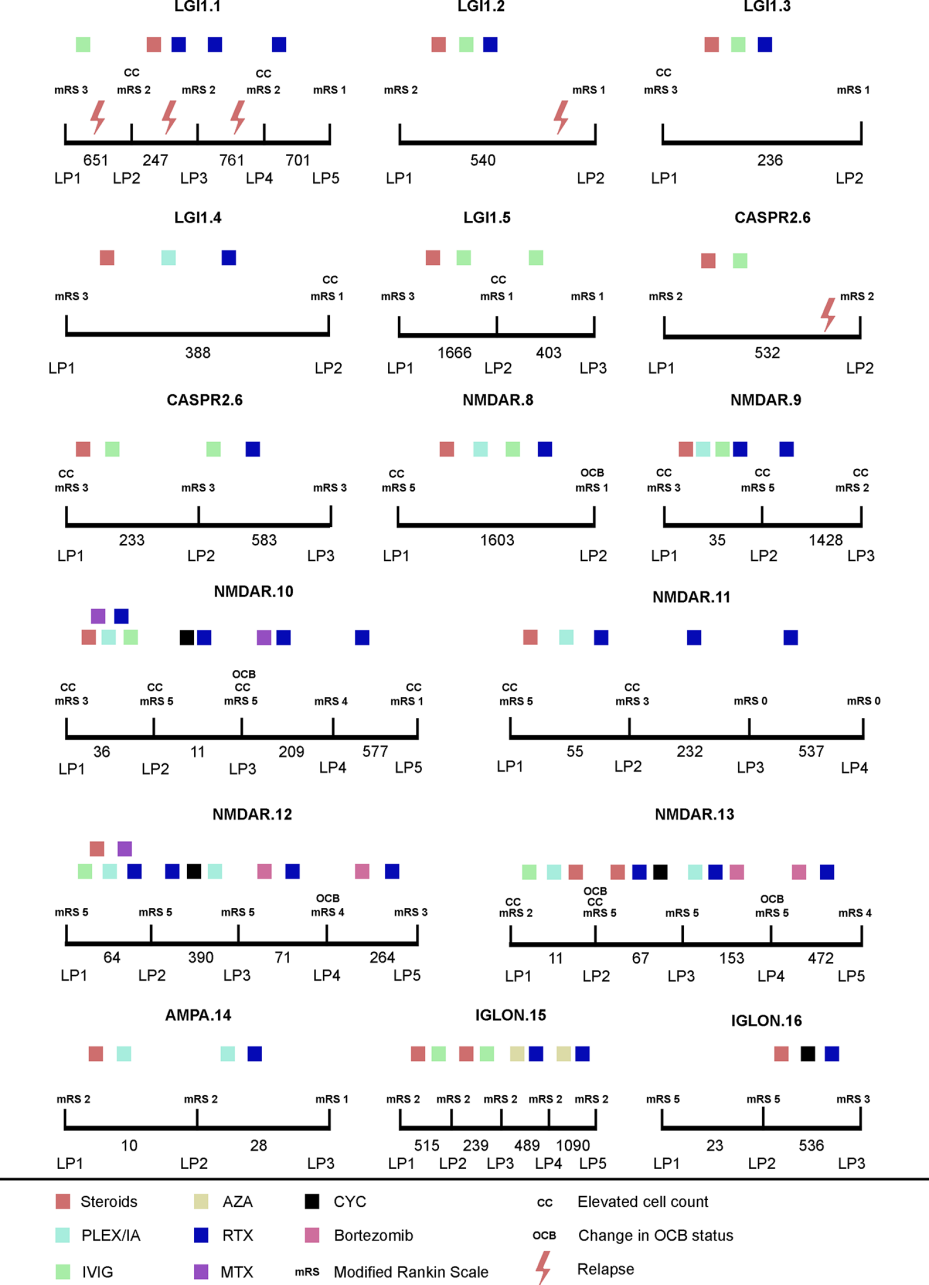
Longitudinal CSF findings and disease course of AIE patients. x axis: time between LP in days; mRS, modified ranking scale; AZA, azathioprine; Bortezomib: bortezomib; CYC, cyclophosphamide; Steroids methylprednisolone; IVIG, intravenous immunoglobulins; MTX, methotrexate; PLEX, plasma exchange; IA, immunoadsorption; RTX, rituximab.

Overall, a trend towards normalization of initial pathological CSF findings was observed over time (**Figure 1** **;** **Table 3**).

**Table 3:** Longitudinal CSF findings.

	LP 1			LP 2				LP 3				LP 4				LP 5			
	CC	*Q*_Alb_	OCB	T	CC	*Q*_Alb_	OCB	T	CC	*Q*_Alb_	OCB	T	CC	*Q*_Alb_	OCB	T	CC	*Q*_Alb_	OCB
**LGI1.1**	1	11.37	–	651	146	28.9	–	247	2	10.35	–	761	26	17.65	–	701	2	11.35	–
	seizures, memory deficits			Memory deficit deterioration, relapse				Memory deficit deterioration, MRI progression, relapse				Seizures, relapse				Stable condition, therapy decision			
**LGI1.2**	3	12.32	–	540	2	12.14	–												
	seizures, memory deficits			Memory deficit deterioration, relapse															
**LGI1.3**	7	2.77	–	236	4	3	–												
	psychosis			Stable condition, therapy decision															
**LGI1.4**	4	6.89	–	388	8	10	–												
	seizures, memory deficits			Stable condition, therapy decision															
**LGI1.5**	3	8.29	–	1666	6	9.4	–	403	2	9.53	–								
	seizures, psychosis, cognitive impairment			Deterioration of cognitive impairment, MRI progression, seizure, relapse				Stable condition, therapy decision											
**CASPR2.6**	3	5.76	–	532	2	5.99	–												
	seizures			Seizure exacerbation, relapse															
**GAD65/67.7**	9	5.81	+	223	6	5.01	+	583	4	6.46	+								
	gait instability, seizure			Progress of gait instability, diplopia, amnesia				Deterioration of the gait instability											
**NMDAR.8**	41	6.65	+	1603	0	3.47	–												
	Psychosis, seizures,			Stable condition, therapy decision															
**NMDAR.9**	64	2.81	+	35	5	4.46	+	1428	6	3.41	+								
	Psychoss			ICU, intractable seizures				Stable condition, therapy decision											
**NMDAR.10**	115	5.96	+	36	85	8.01	+	11	12	22.98	–	209	2	6.84	–	577	9	3.49	–
	Psychosis, seizures,			Intractable seizures, ICU				ICU, clinical improvement, patient can follow simple instructions				general ward, further clinical improvement				Stable condition, therapy decision			
**NMDAR.11**	6	2.8	–	55	8	3.57	–	232	2	2.98	–	537	1	3.77	–				
	psychosis, catatonia, ICU			general ward, clinical improvement				Stable condition, therapy decision				Stable condition, therapy decision							
**NMDAR.12**	2	2.17	+	64	2	5.66	+	390	1	2.28	+	71	0	2.86	–	264	1	4.18	–
	psychosis, seizures, ICU			Coma, ICU				general ward, minimally conscious state				general ward, clinical improvement				Discharge from hospital			
**NMDAR.13**	64	6.38	–	11	106	6.93	+	67	4	10.2	+	153	0	12.14	–	472	4	10.66	–
	psychosis, fever, ICU, intractable seizures			Coma, ICU				Coma, ICU				general ward, minimally conscious state				general ward, marked clinical improvement, discharge from hospital			
**AMPA.14**	2	5.25	–	10	3	2.31	–	28	3	6.36	–								
	Global amnesia			Global amnesia, Reevaluation				Minimal memory deficits, marked clinical improvement											
**IgLON5.15**	2	7.24	–	515	2	4.81	–	239	5	6.87	–	489	4	9.43	–	1090	2	6.7	–
	Depression, memory deficits, EEG/MRI abnormal			Progression of cognitive impairment, Reevaluation				Cognitive impairment, REM sleep disorder				Cognitive decline				subtle cognitive improvement,			
**IgLON5.16**	3	8.2	–	23	2	8.38	–	536	1	7.28	–								
	Vocal cord palsy, tracheostomy, coma of unknown origin			Vocal cord palsy, tracheostoma, coma				Vocal cord palsy, tracheostoma, Cognitive impairment											

CC, cell count per µL; TP, total protein in mg/dl; Alb, Albumin per mg/dl; Q_Alb_ Quotient Albumin serum/CSF; OCB, oligoclonal bands; T, time interval from last LP in days; ICU, intensive care unit.

In patients with anti-LGI1 and anti-CASPR2-abs, 5 LPs were performed during relapse and 4 during stable conditions. Increased cell counts were observed in 2/5 and increased *Q*_Alb_ was found in 4/5 LPs during relapse. During stable conditions, cell counts were increased in 2/4 and *Q*_Alb_ in 3/4 LPs. In anti-NMDAR AIE patients, increased cell counts were observed in 2/7 LPs and increased *Q*_Alb_ was found in 1/7 LPs during stable condition. No relapses were observed; increased cell counts were observed in 5/6 LPs and *Q*_Alb_ was increased in 3/6 LPs at disease onset (**Figure 1**, **Table 3**).

In the largest sub-cohort, namely the anti-NMDAR AIE patients, the OCB status changed during subsequent lumbar punctures. In total, 5 out of 6 anti-NMDAR AIE patients showed positive OCBs during the course of the disease; at the last evaluation time point, only one patient still had positive OCBs. One anti-NMDAR AIE patient, who initially did not show intrathecal immunoglobulin synthesis, consecutively developed positive OCBs (after 11 days). As soon as OCB conversion was evident in anti-NMDAR AIE patients, a temporally associated clinical improvement was noted, e.g., change of consciousness, modified ranking scale (mR) improvement, transfer from intensive care unit (ICU). In anti-NMDAR AIE patients receiving bortezomib, OCBs were absent in the subsequent LP after bortezomib administration and further clinical improvement was noted.

The only patient (NMDAR.11) without any detectable OCBs during the course of the disease and follow-up exhibited an excellent outcome (mRS 0) despite the initial need for ICU admission. In contrast, NMDAR.10, who displayed positive OCBs throughout all follow-ups, exhibited a mild cognitive impairment at last follow-up. Interestingly, in both patients, anti-NMDAR antibody titers were negative. Yet NMDAR.10 did show a signal at the limit of detectability in the cell-based assay in the CSF, while the tissue-based assay remained negative.

## Discussion

CSF analysis is a routine examination in the workup of patients with suspected AIE. In our study, we present CSF findings of a monocentric cohort of patients with confirmed AIE. Our study revealed that in the total cohort, 45.4% of patients had a pleocytosis (64% in the iAIE, 36.6% in the sAIE group) and 60.6% of patients (54.5% in the iAIE, 63.3% in the sAIE group) had elevated total protein levels in the CSF at the time of initial diagnostic workup. When CSF-specific positive OCBs were present, they were usually present at baseline. Over time, the majority of AIE associated with anti-NMDAR patients converted OCB-negative, which coincided with clinical improvement.

Pleocytosis, CSF-specific OCBs or elevated CSF-IgG-indices are key parameters in the diagnostic workup of AIE, with pleocytosis being a core feature to establish the diagnosis AIE (1). In line with a previous cohort study, our data showed that pleocytosis occurs in only half of AIE patients; however, this could also be biased by the fact that the median time from onset to CSF analysis was rather long (11). A recent meta-analysis of CSF findings in AIE indicates AIE subtype-specific differences in inflammatory CSF changes. Higher rates of pleocytosis and positive OCBs in AIE associated with anti-NMDAR, anti-GABABR and anti-AMPAR antibodies were seen in contrast to AIE associated with anti-LGI1, anti-IgLON5 and anti-CASPR2 antibodies (6). In general, albeit constrained by the limited number of patients in each subgroup, we were able to confirm these data with patients suffering from anti-NMDAR AIE, who showed highest increased cell counts and most frequently presented with positive OCB, in contrast to anti-LGI1 or anti-IgLON5 AIE. Although a prior study indicated an age-related influence on the presence of inflammation in the CSF in sAIE patients, no trends were observed in our relatively small sample sized study. Therefore, data have to be reviewed carefully (7).

A previous study investigating CSF parameters in AIE showed pleocytosis in 59% of sAIE patients and 60% of iAIE patients; elevated TP was observed in 37% and 53%, respectively (11). These results are broadly consistent with the data presented here, although the sAIE group was more likely to have normal CSF, possibly due to the higher proportion of anti-LGI1 AIE patients in this cohort (12). However, in line with our data they report that 14% of patients with AIE did not show any abnormalities in CSF analysis (pleocytosis, elevated protein, and OCB) (11). Therefore, when evaluating a patient with suspected AIE, the absence of an abnormal CSF should not lead to prompt rejection of further antibody testing.

Interestingly, the intrathecal immunoglobulins in the CSF were mainly of the IgG isotype, with no evidence of IgM production. This could mean that class-switching has already been completed by the time the first symptoms appear. Also, in the case of surface antibodies e.g. anti-NMDAR, only the pathogenicity of the IgG isotype has been confirmed yet.

In repeated CSF analyses in AIE patients, we observed a trend towards a normalization of cell counts and total protein levels, when condition was clinically stable.

As a novel finding, our study shows that OCBs are transient in most patients with NMDAR AIE and we observed that clinical improvement did coincide with the disappearance of OCBs. Also, negative OCBs during the whole disease course, as seen in one patient, were associated with an excellent outcome. All of the reported NMDAR AIE patients received first and second-line therapy affecting B cells; interestingly, patients receiving bortezomib, which is implicated in targeting plasma cells directly (13), displayed subsequent negative OCB status with clinical improvement. This is in line with previous data reported by Scheibe et al. showing that half of the patients with NMDAR AIE receiving bortezomib developed subsequent negative OCB status (14).

Given the fact that there is evidence that intrathecal plasma cells can produce pathogenic anti-NMDAR antibodies (15) along with autopsy studies showing that plasma cells/plasmablasts are identified in perivascular, interstitial spaces as well as infiltrating the brain parenchyma (16), it is intriguing to speculate that OCBs in anti-NMDAR AIE are disease-specific and could serve as feasible biomarker, which could complement clinical and para-clinical assessments. This is further supported by the fact that a decrease in CSF antibody titers in anti-NMDAR AIE is associated with an beneficial outcome (17). However, as this is purely speculative to date, further studies are necessary to investigate OCBs as a biomarker in anti-NMDAR AIE.

Our study has several limitations: the retrospective analyses of data collected in clinical routine generate a variety of possible biases due to the nature of the study design. A major limitation of this monocentric study is the small sample size within subgroups due to the low prevalence of AIE, which may have limited our conclusions and contributed to the exploratory nature of this study.

In conclusion, we show that in routine CSF analysis a considerable percentage (around 10%) of AIE patients showed normal cell counts or protein levels.

Longitudinally, initial pathologic CSF findings showed a trend toward normalization and change in OCB status in anti-NMDAR AIE, was accompanied by clinical improvement. Further studies would be useful to assess the value of routine CSF analysis in relation to the prognostic value of disease progression both in the initial phase and during follow-ups.

## Data Availability Statement

The original contributions presented in the study are included in the article/supplementary material. Further inquiries can be directed to the corresponding author.

## Ethics Statement

The studies involving human participants were reviewed and approved by Ethics committee of the Medical University of Vienna (EK 1773/2016; 1123/2015). Written informed consent for participation was not required for this study in accordance with the national legislation and the institutional requirements.

## Author Contributions

TZ: study concept and design, patient recruitment, acquisition of data, statistical analysis and interpretation of data, drafting of manuscript. RH: study concept and design, patient recruitment, acquisition of data, statistical analysis and interpretation of data, drafting of manuscript. IW: study concept and design, patient recruitment, acquisition of data, statistical analysis and interpretation of data, drafting of manuscript. TB: study concept and design, patient recruitment, acquisition of data, interpretation of data, critical revision of manuscript for intellectual content, study supervision PR: study concept and design, patient recruitment, interpretation of data, critical revision of manuscript for intellectual content, study supervision. SM: study concept and design, patient recruitment, acquisition of data, statistical analysis and interpretation of data, drafting of manuscript. All authors contributed to the article and approved the submitted version.

## Conflict of Interest

The authors declare that the research was conducted in the absence of any commercial or financial relationships that could be construed as a potential conflict of interest.
